# Isolation and Characterization of a New Phage Infecting *Elizabethkingia anophelis* and Evaluation of Its Therapeutic Efficacy *in vitro* and *in vivo*

**DOI:** 10.3389/fmicb.2020.00728

**Published:** 2020-05-13

**Authors:** Shih-Yi Peng, Li-Kuang Chen, Wen-Jui Wu, Prajna Paramita, Po-Wei Yang, Yun-Zhong Li, Meng-Jiun Lai, Kai-Chih Chang

**Affiliations:** ^1^Department of Laboratory Medicine and Biotechnology, Tzu Chi University, Hualien, Taiwan; ^2^Department of Biochemistry, Tzu Chi University, Hualien, Taiwan; ^3^Department of Clinic Pathology, Buddhist Tzu Chi General Hospital, Hualien, Taiwan; ^4^Department of Laboratory Medicine, Buddhist Tzu Chi General Hospital, Hualien, Taiwan

**Keywords:** *Elizabethkingia anophelis*, bacteriophage, phage therapy, genome analysis, mouse model

## Abstract

*Elizabethkingia* spp. are a group of non-fermentative, Gram-negative, catalase-positive, and non-motile bacilli. They can cause meningitis in neonates and immunosuppressed patients, and lead to high mortality. Considering the rising trend of drug resistance among bacteria pathogens, bacteriophage (phage) therapy is a potential alternative to antibiotics for treating multidrug-resistant bacterial infections. However, so far, no phages specific for *Elizabethkingia* spp. have been reported. Using a clinically isolated *Elizabethkingia anophelis* as the host, the phage TCUEAP1 was isolated from wastewater of Hualien Tzu Chi hospital. The phage particle of TCUEAP1 under electron microscopy was revealed to belong to the siphoviridae family, with a head size of 47 nm, and a tail dimension 12 nm in diameter and 172 nm in length. The one-step growth analysis showed that the latent period of TCUEAP1 was about 40 min with a rise period lasting about 20 min, yielding a burst size of approximately 10 PFU/cell. The adsorption rate of TCUEAP1 reached about 70% in 20 min. Using 20 isolates of *Elizabethkingia* spp. to test the host range of TCUEAP1, it displayed narrow spectrum infecting three strains of *E. anophelis*, but forming spot lysis on 16 strains. The sequence result showed that the genome of TCUEAP1 is a double-stranded DNA of 49,816 bp, containing 73 predicted open reading frames. Further genomic analysis showed TCUEAP1 to be a new phage with no resemblance to publicly available phage genomes. Finally, in a mouse intraperitoneal infection model, at 6 h after the bacterial injection, TCUEAP1 decreased the bacterial load by fivefold in blood. Also, TCUEAP1 rescued 80% of mice heavily infected with *E. anophelis* from lethal bacteremia. We hope that the isolation and characterization of TCUEAP1, the first phage infecting *Elizabethkingia* spp., can promote more studies of the phages targeting this newly emerging bacterial pathogen.

## Introduction

The genus *Elizabethkingia* comprises Gram-negative, non-fermenting, non-motile, obligate aerobic bacteria in the family *Flavobacteriacea*e. This genus was originally designated *Flavobacterium meningoseptica* by King (1959). It was subsequently reclassified into the genus *Chryseobacterium* in 1994 (Vandamme et al., 1994). In 2005, based mainly on 16S rRNA gene sequencing, it was moved to the new genus *Elizabethkingia* (Kim et al., 2005). Genome mapping has enabled further identification of species belonging to this genus, including the *E. meningoseptica*, *Elizabethkingia anophelis*, and *E. miricola* cluster. Notably, accumulating evidence indicates that what previously have been identified as *E. meningoseptica* strains causing sporadic cases of meningitis and bacteremia are actually *E. anophelis* (Coyle, 2017; Han et al., 2017).

*Elizabethkingia anophelis* has been reported to cause neonatal meningitis (Frank et al., 2013), bacteremia (Lau et al., 2016), and nosocomial outbreaks (Teo et al., 2013). Most studies investigating the antimicrobial resistance of *E. meningoseptica* were performed before the proposal of *E. anophelis*; therefore, these studies may actually represent the antimicrobial susceptibility patterns of all *Elizabethkingia* species, particularly *E. anophelis*. Several studies applied reliable species identification methods, and have demonstrated that *E. anophelis* isolates were resistant to most β-lactams, β-lactam/β-lactam inhibitor combinations, carbapenems, and aminoglycosides (Perrin et al., 2017; Lin et al., 2018, 2019; Cheng et al., 2019). A wide variation exists in the susceptibility of *E. anophelis* to piperacillin, piperacillin-tazobactam, ciprofloxacin, levofloxacin, trimethoprim-sulfamethoxazole, and vancomycin (Lin et al., 2019). The treatment options for *E. anophelis* infections are becoming increasingly limited (Janda and Lopez, 2017). For these reasons, there is an urgent need to develop new antibacterial agents to combat the infections caused by *Elizabethkingia* species.

Bacteriophages (phages) are attractive alternatives to antibiotics for treating bacterial infections (Caflisch and Patel, 2019). They have a low environmental impact compared to chemical antibiotics due to their natural origin (Wittebole et al., 2014). They target both Gram-positive and Gram-negative bacteria, and many *in vitro* and *in vivo* models have shown that phages are effective against multidrug-resistant bacteria (Schooley et al., 2017; Dedrick et al., 2019). As antibiotic resistance grows, phages retain the ability to kill antibiotic-resistant bacteria due to their differing mechanisms of action (Abedon et al., 2017). They are highly specific, lysing only their bacterial hosts, and are auto “dosing” in that phage replication at the site of infection, leading to marked increases in their titer (Merril et al., 2003; Abedon et al., 2017). So far, there are no reports of isolation of the phages infecting *Elizabethkingia* species. In this study, we present the isolation and characterization of the world’s first strain of the virulent phage against *E. anophelis*, termed TCUEAP1, from the wastewater of Hualien Tzu Chi hospital. The *in vitro* and *in vivo* therapeutic efficacy of phage TCUEAP1 was also evaluated.

## Materials and Methods

### Bacterial Strains and Growth Conditions

The bacteria used in this study are summarized in Table 1. Among the 20 strains of the *Elizabethkingia* species, the reference strain of *E. meningoseptica* was obtained from the Bioresource Collection and Research Center (BCRC), and the other 19 non-repetitive isolates were collected from two branches (Hualien and Taipei) of the Buddhist Tzu Chi General Hospital in Taiwan. The species were initially identified using the Vitek 2 compact system (Biomerieux, France). Further differentiation of these isolates as *E. meningoseptica* or *E. anophelis* was performed using PCR, based on the method described by Chew et al. (2018). When isolates could not be identified as *E. meningoseptica* or *E. anophelis* by PCR, sequencing of the 16S rRNA gene was used as the reference standard for species identification (Chang et al., 2014). To analyze the homology of the collected strains, pulsed-field gel electrophoresis (PFGE) was performed (Chang et al., 2014). Bacteria were cultivated in Luria Bertani (LB) broth or LB agar (BioShop Canada Inc., Burlington) at 25 or 37°C. The growth of bacteria was monitored by measuring the optical density at 600 nm (OD_600_), where an OD unit of 1.0 corresponded to 1 × 10^9^ cells/ml.

**TABLE 1:** Bacterial strains used to screen the phages from wastewater and to perform the host range analysis of TCUEAP1.

**No.**	**Bacterial strain**	**Plaque formation^a^**	**TCUEAP1 spot test^b^**	**Source**
***E. meningoseptica***				
1	*E. meningoseptica* BCRC 10677	-	N	BCRC
2	*E. meningoseptica* MEN1	-	P	Clinical isolate from Buddhist Tzu Chi General Hospital (BTCGH), Hualien
***E. anophelis***				
3	*E. anophelis* ANO1	−	P	Clinical isolates from BTCGH, Hualien
4	*E. anophelis* ANO2	−	P	
5	*E. anophelis* ANO3	−	P	
6	*E. anophelis* ANO5	−	P	
7	*E. anophelis* ANO9	−	P	
8	*E. anophelis* ANO10	+	P	
9	*E. anophelis* ANO12	+	P	
10	*E. anophelis* ANO13	−	P	
11	*E. anophelis* ANO14	−	P	
12	*E. anophelis* ANO15	+	P	
13	*E. anophelis* ANO16	−	N	
14	*E. anophelis* ANO17	−	P	
15	*E. anophelis* ANO18	−	P	
16	*E. anophelis* ANO19	−	P	Clinical isolate from BTCGH, Taipei
17	*E. anophelis* ANO20	−	P	
18	*E. anophelis*eANO21	−	P	
***E. miricola***				
19	*E. miricola* MIR1	−	N	Clinical isolates from BTCGH, Taipei
20	*E. miricola* MIR2	−	N	

*^*a*^**+**, susceptibility to TCUEAP1; **−**, no susceptibility to TCUEAP1.^*b*^**P**, clear spot could be seen; **N**, no clear spot could be seen.*

### Isolation and Purification of Bacteriophage

All the wastewater samples were collected in Hualien Tzu Chi hospital and were used for screening phages specific to the isolated *Elizabethkingia* strains. Thirty milliliters of wastewater samples were centrifuged at 10,000 × *g* for 10 min to clear bacteria and debris, and filtered through a 0.22-μm Millipore filter. To enrich the phages in the wastewater samples, each time 25 ml of filtrate was mixed with 500 μl of bacterial solution consisting of five *Elizabethkingia* strains (10^8^ CFU each) all in log-phase and incubated at 37°C for 4 h. In total, 20 strains were used for the enrichment procedure. After the enrichment period, the mixtures were centrifuged at 10,000 × *g* for 10 min and filtered through 0.22-μm Millipore filters. For each tested bacterial strain, 1 ml of the filtrate was mixed with 100 μl of bacteria culture (10^8^ CFU) in log phase. The mixture was added to 5 ml of soft LB agar (0.7%) and then poured over solidified LB agar plates. The plates were incubated overnight at 25°C. For the enriched samples showing inhibited bacterial growth, the clear zone of the single plaque was picked using a sterile Pasteur pipette tip, and propagated in a new culture. The new mixture was serially diluted and plated on soft agar medium (0.7%) again for plaque formation. This procedure was repeated three times for phage purification.

### Phage Purification

Lysates for TCUEAP1 phage purification were prepared by infecting 200 ml of *E. anophelis* ANO15 in early log-phase with TCUEAP1 at a multiplicity of infection (MOI) of about 1.0, and incubating with aeration for 4 h. Crude lysates were centrifuged and the supernatants were passed through a membrane filter with a pore size of 0.45 μm. The phage particles were concentrated by centrifugation for 2 h at 39,000 × *g* in a Beckman Avanti J-25I. The pellets were re-suspended in 1.0 ml of TE buffer and purified by banding on the block gradient of CsCl representing 1.2, 1.3, and 1.4 g/cm^3^ (2 ml for each block) in ultracentrifugation. The ultracentrifugation conditions were 107,200 × *g* for 3 h at 4°C with the SW41Ti rotor in a Beckman LE-80K. The phage band collected was dialyzed against the TE buffer and then stored at 4°C until further use.

#### Transmission Electron Microscopy (TEM) of Phage Particles

Phage morphology was examined by TEM with negatively stained preparations. A drop of purified phages with a titer of approximately 10^10^ PFU/ml was applied to the surface of a formvar-coated grid (200-mesh copper grids), followed by negative staining with 2% uranyl-acetate (pH 3). The phage morphology was examined by using a Hitachi H-7500 transmission electron microscope operated at 80 kV (Hitachi Company, Japan).

### Phage DNA Preparation, Genome Sequencing, and Annotation

DNA of the purified phage TCUEAP1 was prepared by extraction with phenol/chloroform and ethanol precipitation. The genome was sequenced by MiSeq (Illumina, Inc., San Diego, CA, United States). Raw reads were assembled using Bowtie (v1.1.1) (Langmead et al., 2009). The termini and package mode of the genome were analyzed by PhageTerm (Garneau et al., 2017). Putative protein-encoding open reading frames (ORFs) on the resulting whole genome were predicted using the program Prodigal (Hyatt et al., 2010). The functions of the proteins were annotated by running NCBI protein BLAST^1^ against *nr* database. Further annotations were helped by restricting the BLAST search to the organism *virus* (taxid 10239). The genomic sequence of phage TCUEAP1 has been submitted to NCBI (GenBank accession number MN732896).

### Determination of Optimal MOI

Host strain *E. anophelis* ANO15 was grown in LB medium at 37°C to the early log phase (10^8^ CFU/ml). Six different ratios of TCUEAP1 to the host strain were added to LB medium, for MOI 0.001, 0.01, 0.1, 1, 10, and 100 PFU/CFU. After 3.5 h of incubation at 25 or 37°C, the samples were collected, and the titers of the phages were determined by serial dilution and the double-layer method (Adams, 1959). All experiments were performed in triplicate.

### *In vitro* Adsorption Assay

The adsorption experiment was performed according to Adams (1959). When the growth of the host strain *E. anophelis* ANO15 and the control strain *E. meningoseptica* BCRC 10677 reached an OD_600_ of 1.0, 2 ml of each culture was harvested and fresh LB broth was added to a final volume of 20 ml. Phage stock of TCUEAP1 was added to each diluted culture at an MOI of 1 and incubated at 25°C for 30 min. Aliquots of 100-μl samples were collected from 0 to 30 min at 5-min intervals, and centrifuged at 10,000 *g* for 10 min. The supernatants containing the non-adsorbed phages were titrated by serial dilution and the double-layer method. The percentage of free phages was calculated by dividing the phage titer in the supernatant to that in the initial phage stock. All experiments were performed in triplicate.

### One-Step Growth Curve

The culture of the host strain was incubated to an OD_600_ of 1.0. Three-milliliter aliquots of the bacterial culture were mixed with the phage TCUEAP1 at an MOI of 1, and the phages were allowed to adsorb to the host for 10 min at 25°C. The mixture was centrifuged, and the supernatant was discarded to remove free phage particles. The pellets were re-suspended in 30 ml of fresh LB broth and incubated at 25°C. Two sets of the samples were collected every 10 min, for 80 min. One of the two samples was treated with 1% chloroform (final concentration) to release intracellular phages. Then, the sets of the samples were immediately titrated by serial dilution and the double-layer method. All experiments were performed in triplicate. The latent period, eclipse period, and burst size of TCUEAP1 were derived from the growth curve.

### Phage Infection Assay

The culture of the host strain *E. anophelis* ANO15 was incubated to an OD_600_ of 0.5 and then was mixed with the phage TCUEAP1 at different MOIs (from 0.0001 to 10) at 25 or 37°C. The control experiments were performed using equal volume of phage buffer [10 mM Tris-HCl (pH 7.5), 10 mM MgSO_4_, 68.5 mM NaCl, and 1 mM CaCl_2_] to replace the phage stock. The post-infection OD_600_ values were measured for 14 h, at 30-min intervals for the first 7 or 8 h.

### Host Range Analysis

The host range of TCUEAP1 was examined by both the double-layer method (Adams, 1959) and the spot test (Romig and Brodetsky, 1961) on the 20 *Elizabethkingia* spp. used to screen phages from the water samples (Table 1). The bacterial strains were separately cultured to reach an OD_600_ of 1. For the double-layer method, 100 μl of 10-fold serially diluted (10^1^ to 10^9^) phage stock was mixed with 100 μl of the bacterial culture and 5 ml of soft LB agar (0.7%) and then poured over the solid LB agar plates. Plates were incubated overnight at 25°C. For the spot test, 100 μl of the bacterial culture was included in the top agar of the double-layered agar plates, and 10 μl of phage-containing solution (10^5^ PFU) was spotted onto the bacterial lawns. Plates were dried in a laminar flow hood for 10 min and incubated at 25°C for 18 to 20 h. The observation of plaques on the bacterial lawn was recorded. Each test was repeated three times.

### Effect of Temperature and pH on TCUEAP1 Stability

Thermal and pH stability tests were carried out using the double-layer method (Adams, 1959). In the thermal stability tests, aliquots of phage TCUEAP1 preparations (10^9^ CFU/ml) were treated at pH 7 and different temperatures (4, 25, 37, 50, 60, and 70°C) in distilled water for 1 h. For the pH stability experiments, phage preparations were treated with various pH buffers (pH 2, 4, 7, and 11) at 25°C in distilled water for 1 h. The number of the remaining infective phages was determined by the double-layer method at 25°C.

### Phage Therapy in the Mouse Sepsis Model

To evaluate the ability of phage TCUEAP1 to control the bacterial load and prevent lethal bacteremia, three experimental conditions were tested. Thirty eight-week-old male BALB/c mice (provided by NLAC Company, Taiwan) were randomly assigned to three groups (*n* = 10 per group). *E. anophelis* ANO15 was grown in LB broth at 37°C for 18 h and adjusted to the appropriate concentration (1 × 10^10^ CFU/ml) in normal saline (0.9% NaCl). The first group was only inoculated intraperitoneally with 100 μl of normal saline and phage TCUEAP1 (1 × 10^9^ PFU/mouse, 100 μl/mouse) to assess the phage toxicity to mice. The remaining two groups were inoculated intraperitoneally with 1 × 10^9^ CFU of *E. anophelis* ANO15 (1 × 10^9^ CFU/mouse, 100 μl/mouse), and 2 h later, intraperitoneal administration with either phage TCUEAP1 (1 × 10^9^ PFU/mouse, 100 μl/mouse) or 100 μl of normal saline. After 6 h post-infection, seven mice were randomly selected from each of the two groups and 50 μl of blood was taken by tail vein sampling to evaluate the bacterial load. The animals were monitored for 7 days and the survival rate of each group was calculated.

### Statistical Analysis

Data are expressed as the mean ± standard deviation. The statistical significance of all the data was analyzed with the Student’s *t*-test. Statistical significance was defined as *p* < 0.05. Statistical analysis of the mouse survival rate evaluations was performed using GraphPad Prism software 6.0 with *p* < 0.05.

### Ethics Statement

All experiments involving the mice and all the care and handling of the mice were performed using protocols approved by the Institutional Animal Care and Use Committee of Tzu Chi University (No. 107083). All methods were performed under the relevant guidelines.

## Results

### Isolation of a New Phage for *E. anophelis*

**FIGURE 1:**
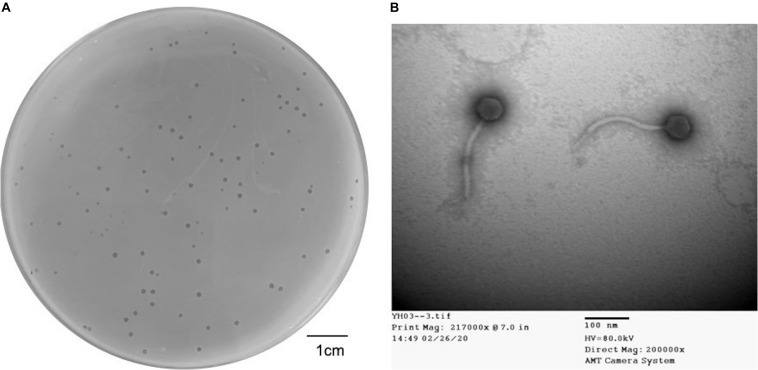
Plaque and transmission electron microscopic (TEM) image of phage TCUEAP1. **(A)** Plaques formed by phage TCUEAP1. Scale bar: 1 cm. **(B)** Morphology of phage TCUEAP1 revealed under TEM. Scale bar: 100 nm.

Dozens of wastewater samples around Hualien Tzu Chi hospital were collected and screened by the double-layer method using 20 strains of *Elizabethkingia* spp. One obtained phage that formed clear and haloed round plaques of about 1–2 mm in diameter (Figure 1A) on *E. anophelis* ANO15 was designated as TCUEAP1. TEM showed that TCUEAP1 possessed an icosahedral head with a diameter of approximately 47 ± 3 nm, and a tail that was 172 ± 3 nm long and 12 ± 1 nm in diameter (Figure 1B). The morphology suggested that TCUEAP1 could be assigned to the order *Caudovirales* and family *Siphoviridae*.

### Optimal MOI of Phage TCUEAP1

**FIGURE 2:**
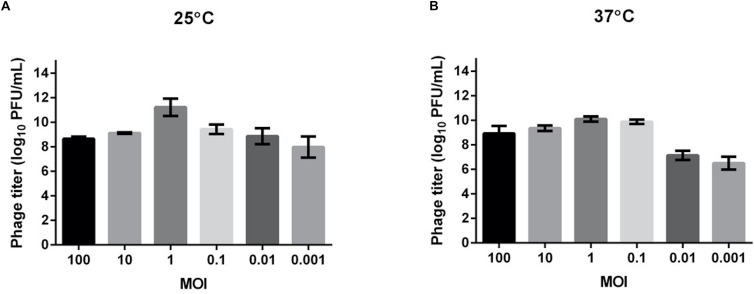
Determination of the optimal multiplicity of infection (MOI) for TCUEAP1. Comparison of the phage titers after 3.5 h of incubation at six MOI values (0.001, 0.01, 0.1, 1, 10, and 100 PFU/CFU) at 25°C **(A)** and 37°C **(B)**. The experiments were repeated three times.

We tested the MOI ranging from 0.001 to 100 to determine the optimal values for producing maximal phage titers at 25 and 37°C (Figure 2). The results showed that at both temperatures, the optimal MOI was 1, at which the phage titer at 25°C (about 10^11^ PFU/ml) was even higher than at 37°C (about 10^10^ PFU/ml). This MOI was used for the following experiments.

### Biological Characterization of TCUEAP1

**FIGURE 3:**
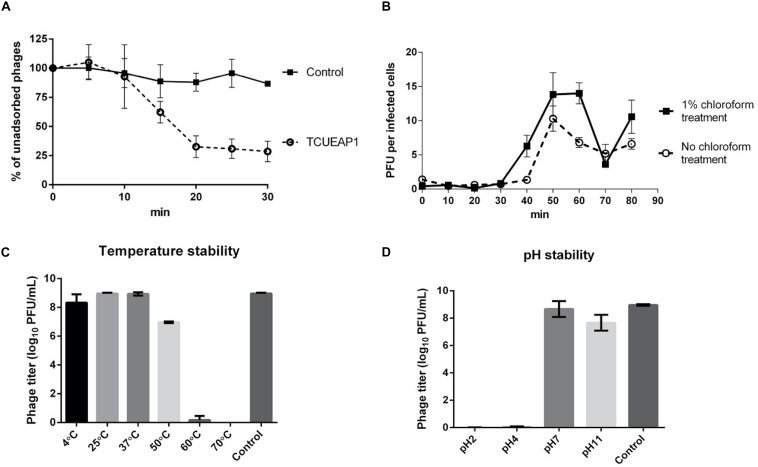
Characterization of TCUEAP1. **(A)** Adsorption curve of the phage TCUEAP1 to the host *E. anophelis* ANO15 and the control *E. meningoseptica* BCRC 10677 at an MOI of 1 at 25°C. Aliquots were taken at intervals of 5 min (up to 30 min), and the supernatant containing the non-adsorbed phages were titrated. Percentage unadsorbed phages is the ratio of the phage titer in the supernatant to that in the initial phage stock. **(B)** One-step growth curve of the phage TCUEAP1 on *E. anophelis* ANO15 at 25°C. PFU per infected cells in chloroform-treated and chloroform-untreated cultures are shown at different time points. **(C)** The effect of temperature. TCUEAP1 was incubated at different temperature values for 1 h before determining the phage titer. **(D)** The effect of pH on TCUEAP1. TCUEAP1 was incubated at different pH values for 1 h before determining the phage titer. All experiments were repeated three times.

The adsorption experiment performed at an MOI of 1 at 25°C showed that approximately 70% of the phage particles of TCUEAP1 were adsorbed to the host *E. anophelis* ANO15 within 20 min of incubation, and no significant rise was observed as the incubation time increased further (Figure 3A). To characterize the infection process of the phage, the one-step growth curve of TCUEAP1 on *E. anophelis* ANO15 at an MOI of 1 at 25°C was determined.

The growth curves revealed that the eclipse and latent period of TCUEAP1 were around 30 and 40 min, respectively. The rise period lasted for about 20 min, and released a small burst size of about 10 PFU per infected cell (Figure 3B). The stability of phage TCUEAP1 at varying temperatures and pH conditions was also investigated. TCUEAP1 phages showed their viability at the tested temperature from 4 to 50°C, maximally at 25 and 37°C, and were quickly inactivated when heated to 60°C (Figure 3C). At pH 7 and 11, phage TCUEAP1 survived generally well, while no titer was recorded at a pH of 2 and 4 (Figure 3D).

### Host Specificity and Host Growth Inhibition of TCUEAP1

**FIGURE 4:**
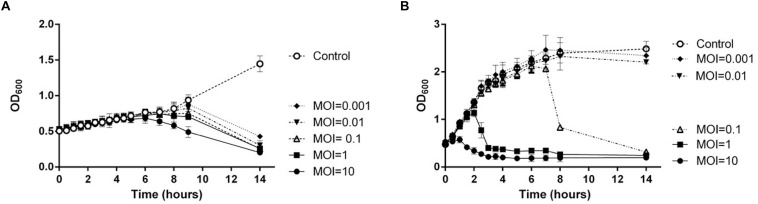
The infection assay of the phage TCUEAP1 against *E. anophelis* ANO15 *in vitro* at 25°C **(A)** and 37°C **(B)**. *E. anophelis* ANO15 was infected by the phage TCUEAP1 at MOI values of 10, 1, 0.1, 0.01, or 0.001, and cultured for 14 h. The control experiments were performed using equal volumes of phage buffer. This experiment was repeated three times.

To evaluate the host specificity of TCUEAP1, phage lysates were tested using the double-layer method and the spot test against 20 strains of *Elizabethkingia* species listed in Table 1. None of them share the same pulsotypes. The results of the double-layer method indicated that TCUEAP1 could only form plaques against three strains of *E. anophelis*, including the host strain *E. anophelis* ANO15. In contrast, the results of the spot test showed that TCUEAP1 solution was able to form clear spots on 16 isolates of *Elizabethkingia* spp., including one *E. meningoseptica* strain (Table 1). The infection assays of phage TCUEAP1 against *E. anophelis* ANO15 at 25 and 37°C were performed *in vitro*. The results (Figure 4) showed that at 25°C, even at MOI 0.001, phage TCUEAP1 could effectively reduce the growth of *E. anophelis* ANO15. In contrast, the results at 37°C showed that when the MOI was under 0.01, TCUEAP1 had no observable effect on the growth of the host.

### Sequencing and Bioinformatics Analysis of TCUEAP1 Genomic DNA

**FIGURE 5:**
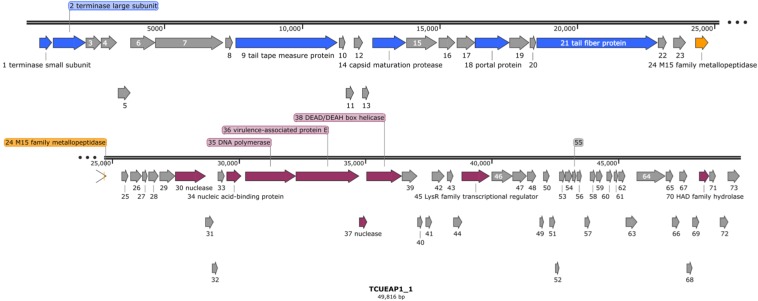
The genomic organization of phage TCUEAP1. Predicted open reading frames (ORFs) are represented as arrows with colors referring to the predicted functions for the derived proteins. Gray color shows hypothetical proteins with unknown functions; blue color, structural proteins; orange color, a possible lysis module; magenta color, replication and regulation-related proteins. The figure was generated using the program SnapGene Viewer (v. 4.3.11) http://www.snapgene.com/.

Raw reads of the genome of TCUEAP1 were assembled into a circularly permutated form. By detecting the biases in the number of sequence reads, PhageTerm (Garneau et al., 2017) software predicted the genome to be a concatemeric DNA packaged through a headful packaging mechanism. The resulting genome of TCUEAP1 is a double-stranded linear DNA with a length of 49,816 bp and GC content of 39.34%. It was predicted by Prodigal (Hyatt et al., 2010) to contain 73 ORFs, all located on the positive strand. No tRNA genes were detected by the tRNAscan-SE 2.0 web server. Through the protein BLAST analysis, only putative products from 21 ORFs could find statistically significant (*E* < 0.001) matches, of which six only matched to hypothetical proteins in the databases. The annotations adopted in this paper were based on the BLAST search against the proteins of virus (taxid) origins (Supplementary Table S1). Overall, annotations (Figure 5) showed that the ORFs predicted to encode structural products were located in the upstream genomic cluster, and the ORFs related to replication and regulation were in the downstream genomic cluster. ORF 24 between the two clusters encodes a putative product containing the M15 metallopeptidase domain, a functional module related to phage endolysin (Mikoulinskaia et al., 2009). No lysogenic characteristics, such as integration-related or CI repressor genes were identified.

### Phage Therapy in the Mouse Sepsis Model

**FIGURE 6:**
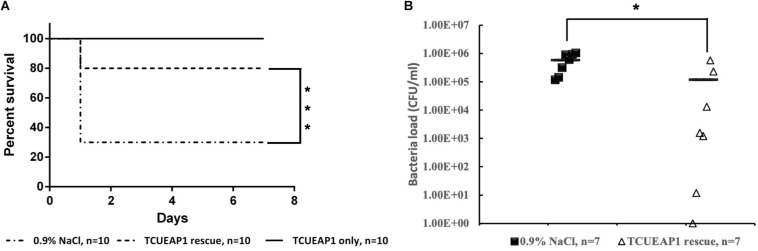
Therapeutic efficacy of phage TCUEAP1 against mice with sepsis induced by *E. anophelis* infection. **(A)** The survival of the mice treated with a single dose of phage TCUEAP1 or normal saline intraperitoneally after *E. anophelis* infection. The survival of the mice was monitored for 7 days. ***p < 0.001 for normal saline versus phage TCUEAP1. **(B)** Blood samples of mice treated with normal saline (solid square) versus those of mice treated with phage TCUEAP1 (open triangle) at 6 h after bacterial inoculation. The line indicates mean bacterial load; *p < 0.05 for normal saline versus phage TCUEAP1.

Since lethal bacteremia is a common outcome of *E. anophelis* infection, we investigated the ability of phage TCUEAP1 to rescue mice from lethal sepsis induced by intraperitoneal injection of *E. anophelis*. The minimum lethal dose of *E. anopheles* to BALB/c mice was determined to be 1 × 10^9^ CFU/mouse (Supplementary Figure S1). To assess the biosafety of phage TCUEAP1, 10 mice were inoculated intraperitoneally with phage TCUEAP1 (1 × 10^9^ PFU/mouse) and monitored for 7 days. The results showed that all mice in the control group had normal vital signs and survived the 7 days of observation period (Figure 6A). Each mouse received 10^9^ CFU of *E. anophelis* intraperitoneally, and was treated 2 h later with a single dose of 10^9^ PFU of phage TCUEAP1 or normal saline by the same route. While 70% of normal saline-treated mice died within 1 day, the phage TCUEAP1-treated mice had a significantly higher rate of survival, with 80% being rescued from the lethal dose of *E. anophelis* (Figure 6A). To quantify the ability of phage TCUEAP1 to reduce *E. anophelis* load *in vivo*, after 6 h post-*E. anophelis* infection, seven mice were randomly selected from each of the two groups to measure the bacterial load in the blood. There was approximately 5 × 10^5^ CFU/ml of bacteria in the blood from the normal saline-treated mice (Figure 6B). In contrast, the bacterial load in the blood collected from the mice treated with phage TCUEAP1 was reduced at least fivefold (*p* < 0.05), to the level about 1 × 10^5^ CFU/ml. These results collectively indicate that phage TCUEAP1 has good antimicrobial activity *in vivo*.

## Discussion

As more studies have employed advanced species-identification methods, the clinical importance of *E. anophelis* has begun to emerge, having been identified as a novel human pathogen in just a few years (Janda and Lopez, 2017). The rising trend of drug resistance among bacterial pathogens prompts a consideration of phage therapy (Gorski et al., 2018; Caflisch and Patel, 2019). Among the flourish of various phage studies, there is no discussion of use against the novel pathogen *Elizabethkingia*. In this work, from dozens of wastewater samples, we isolated TCUEAP1, the virulent phage infecting *E. anophelis*. Characterization and genome analysis of TCUEAP1 were performed.

The stability studies showed that the phage could not resist high temperatures (above 60°C) and was very sensitive to an acidic environment (pH 2 and pH 4). The specificity test using 20 bacterial strains showed that while TCUEAP1 could only infect three strains, the phage lysate had a broad lytic spectrum against 16 strains. The difference may imply the possible existence of phage-encoded products, such as endolysin and hydrolase in the phage lysates. The adsorption rate of TCUEAP1 to the host bacteria could reach 70% in 20 min, but then appeared to encounter a limit and did not change much after further 10 min. As stated earlier, in the optimal MOI and infection assays of TCUEAP1 at 25 and 37°C, TCUEAP1 propagated better at 25°C, and could reduce the growth of the host bacteria even when the MOI was as low as 0.001. This matched our experience when we tried to isolate the phages targeting *Elizabethkingia* from the environment. We found it helpful to perform the co-culture of TCUEAP1 with its host at 25°C to slow down the bacterial growth rate and allow the phage to propagate to a suitable amount.

The genome of phage TCUEAP1 was sequenced and analyzed. For more than 70% (52 out of 73) of possible proteins derived from the TCUEAP1 genome, we could not identify any homologs using BLAST searches. Also, as we tried to identify the evolutionary related phages of TCUEAP1, among the 73 putative protein products, maximally only seven of them (mainly products of ORF 34 to 38) could find possible homologs from a single phage origin by BLAST. This patchy sequence similarity means that the traditional linear phylogenetic relationship is not suitable for TCUEAP1. We then tried to use vConTACT2 (v0.9.15) to assign the taxonomic status of TCUEAP1. vConTACT2 is a network-based application using phage genomes and their protein content for phage classification (Bin Jang et al., 2019). This type of program was regarded to be more suitable for the highly mosaic phenomenon seen on phage genomes (Dion et al., 2020). The analysis indicated TCUEAP1 to be a “singleton” virus, meaning no close relatives could be identified in the phage genome database (ProkaryoticViralRefSeq97-Merged). This means that more viral genome space needs to be sampled to build up the connections of TCUEAP1 to other phages.

The putative product of ORF36 was suggested to be a virulence-associated protein E (vapE) in BLAST results. Apart from one report showing that, in the bacterial pathogen of swine, deleting the vapE gene reduced the bacterial pathogenesis in mice (Ji et al., 2016), no details about the nature of the virulence have been identified so far. Among the BLAST results, there were a few protein hits for the predicted product of ORF36 being annotated as DNA primase (Supplementary Table S1), revealing another possible function for the predicted ORF36.

Endolysin, also termed phage lysin, is the protein encoded by phages to degrade the peptidoglycan of bacteria. It has been suggested to be a potent antibacterial agent separately from the entire phage (Lai et al., 2011). The potential product of ORF24 in the TCUEAP1 genome was identified to contain the M15 family peptidase domain, implying its possible connection to endolysin function. This presumption is currently under investigation.

When applied to the mice with sepsis induced by *E. anophelis*, TCUEAP1 was able to decrease the bacterial load by over fivefold and enhanced mice survival. Considering the new clinical challenges from *Elizabethkingia*, we believe that the isolation and characterization of the world’s first phage against *Elizabethkingia* is valuable. It not only provides a way to understand the *Elizabethkingia* bacteria but can also further the preparation of weapons to combat *Elizabethkingia* infections in the future.

## Data Availability Statement

The datasets generated for this study can be found in the GenBank accession number MN732896.

## Ethics Statement

The animal study was reviewed and approved by Institutional Animal Care and Use Committee of Tzu Chi University (No. 107083).

## Author Contributions

K-CC conceived and guided this project. K-CC and M-JL analyzed the results and wrote the manuscript. S-YP, L-KC, PP, P-WY, and Y-ZL performed the characterization of the phage TCUEAP1. S-YP and W-JW performed the animal studies. All authors reviewed the manuscript.

## Conflict of Interest

The authors declare that the research was conducted in the absence of any commercial or financial relationships that could be construed as a potential conflict of interest.

## Footnotes

1 http://www.ncbi.nlm.nih.gov/
