# Supplementary material for: Isolation and Characterization of a New Phage Infecting Elizabethkingia anophelis and Evaluation of Its Therapeutic Efficacy in vitro and in vivo
Source: Front Microbiol. 2020 May 13;11:728. doi: 10.3389/fmicb.2020.00728 (PMC7237756; doi:10.3389/fmicb.2020.00728)
Supplement: Supplementary file 1 [file Data_Sheet_1.pdf]

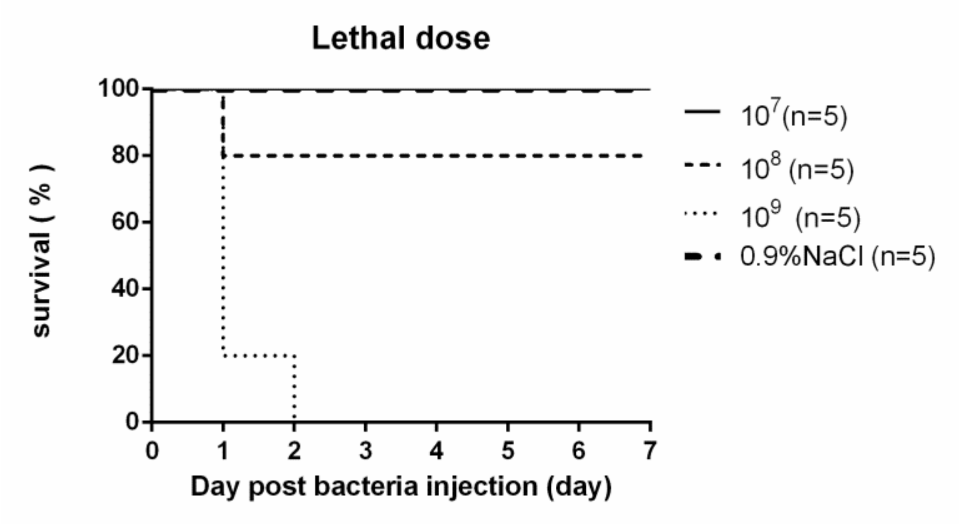

Supplementary FIGURE 1 |The minimum lethal dose (CFU) of *E. anophelis* to the mice with sepsis induced by intraperitoneal injection.
